# Supplementary material for: Microgravity enhances the phenotype of Arabidopsis zigzag-1 and reduces the Wortmannin-induced vacuole fusion in root cells
Source: NPJ Microgravity. 2022 Sep 6;8:38. doi: 10.1038/s41526-022-00226-3 (PMC9445043; doi:10.1038/s41526-022-00226-3)
Supplement: Supplementary file 1 — Supplemental Material [file 41526_2022_226_MOESM1_ESM.pdf]

Microgravity enhances the phenotype of *Arabidopsis zigzag-1* and reduces the Wortmannin-induced vacuole fusion in epidermal root cells

Mengying Wang, Katherine Danz, Vanessa Ly and Marcela Rojas-Pierce

Supplemental Data

Supplementary Table 1. Genotypes and treatments used in the BRIC-PDFU.

BRIC Name	Position of PDFU	Genotype	Treatment	Position of PDFU	Genotype	Treatment
**A**	1	WT	DMSO	4	*zig-1*	DMSO
	2	WT	WM	5	*zig-1*	WM
	3	WT	DMSO	6	WT	WM
**B**	1	WT	DMSO	4	*zig-1*	DMSO
	2	WT	WM	5	*zig-1*	WM
	3	*zig-1*	DMSO	6	HOBO temperature recorder	
**C**	1	WT	DMSO	4	*zig-1*	DMSO
	2	WT	WM	5	*zig-1*	WM
	3	*zig-1*	WM	6	*zig-1*	WM
**D**	1	WT	DMSO	4	*zig-1*	DMSO
	2	WT	WM	5	*zig-1*	WM
	3	*zig-1*	DMSO	6	HOBO temperature recorder	

Supplementary Table 2. Statistics summary for vacuole number counts of cells from specific cell types and cell layers in the root.

Assay type		Ground control				Flight assay			
Treatment		WT DMSO	WT WM	*zig-1* DMSO	*zig-1* WM	WT DMSO	WT WM	*zig-1* DMSO	*zig-1* WM
Epidermis transition	Mean ± Std. Deviation	1.2 ± 0.5	1.1 ± 0.4	2.6 ± 1.1	1.4 ± 0.8	1.2 ± 0.5	1.2 ± 0.6	2.6± 1.4	1.7 ± 1.0
Epidermis elongation	Mean ± Std. Deviation	1.0 ± 0.2	1.0 ± 0.2	3.3 ± 1.5	1.7 ± 1.1	1.1 ± 0.3	1.1 ± 0.3	3.8 ± 2.0	2.9 ± 1.8
Cortex transition	Mean ± Std. Deviation	1.2 ± 0.5	1.2 ± 0.6	2.6 ± 1.0	1.5 ± 0.8	1.2 ± 0.6	1.2 ± 0.5	2.6 ± 1.4	1.6 ± 1.1
Cortex elongation	Mean ± Std. Deviation	1.0 ± 0.1	1.0 ± 0.1	2.5 ± 1.2	1.4 ± 0.8	1.0 ± 0.3	1.0 ± 0.2	2.9 ± 2.1	1.4 ± 1.0

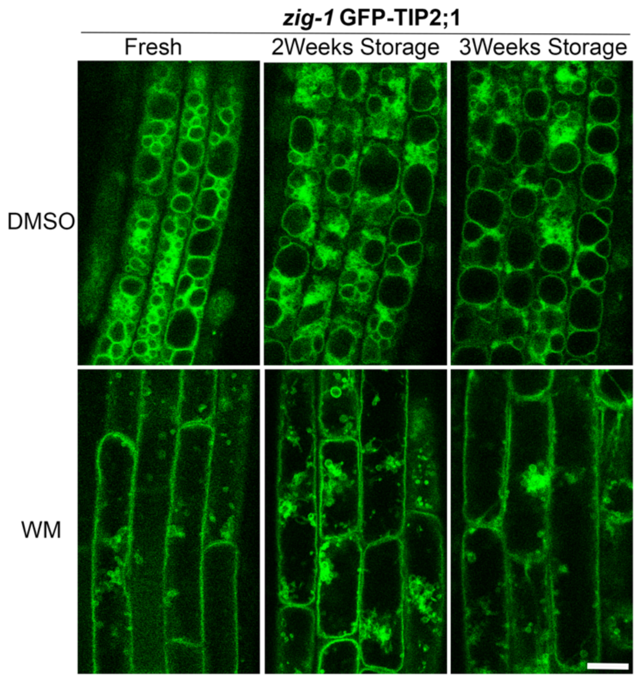

Supplementary Figure 1. DMSO and WM solution are active after 2-3 weeks of storage at 4°C. *zig-1* seedlings expressing GFP-TIP2;1 were treated with fresh DMSO or WM solution and solutions that had been stored for 2-3 weeks. 2-3 weeks-old DMSO did not change cell physiology while 2-3 weeks old WM were still functional to induce vacuole fusion of *zig-1*. Scale bar: 20 μm

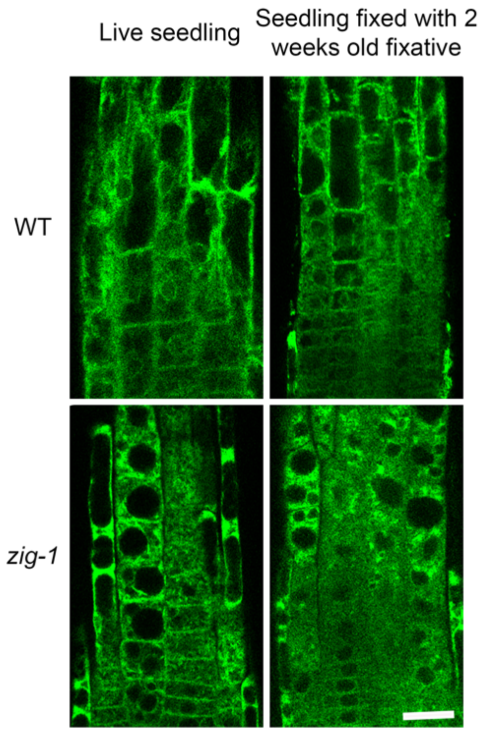

Supplementary figure 2. Fixative containing 4%PFA in 0.5X PBS buffer was active after 2 weeks storage at 4°C.
WT and *zig-1* seedlings expressing GFP-TIP2;1 were fixed with fixative solution after 2 weeks of storage at 4°C. Cell morphology of stored fixative was similar to fresh seedlings.
Scale bar: 20 μm

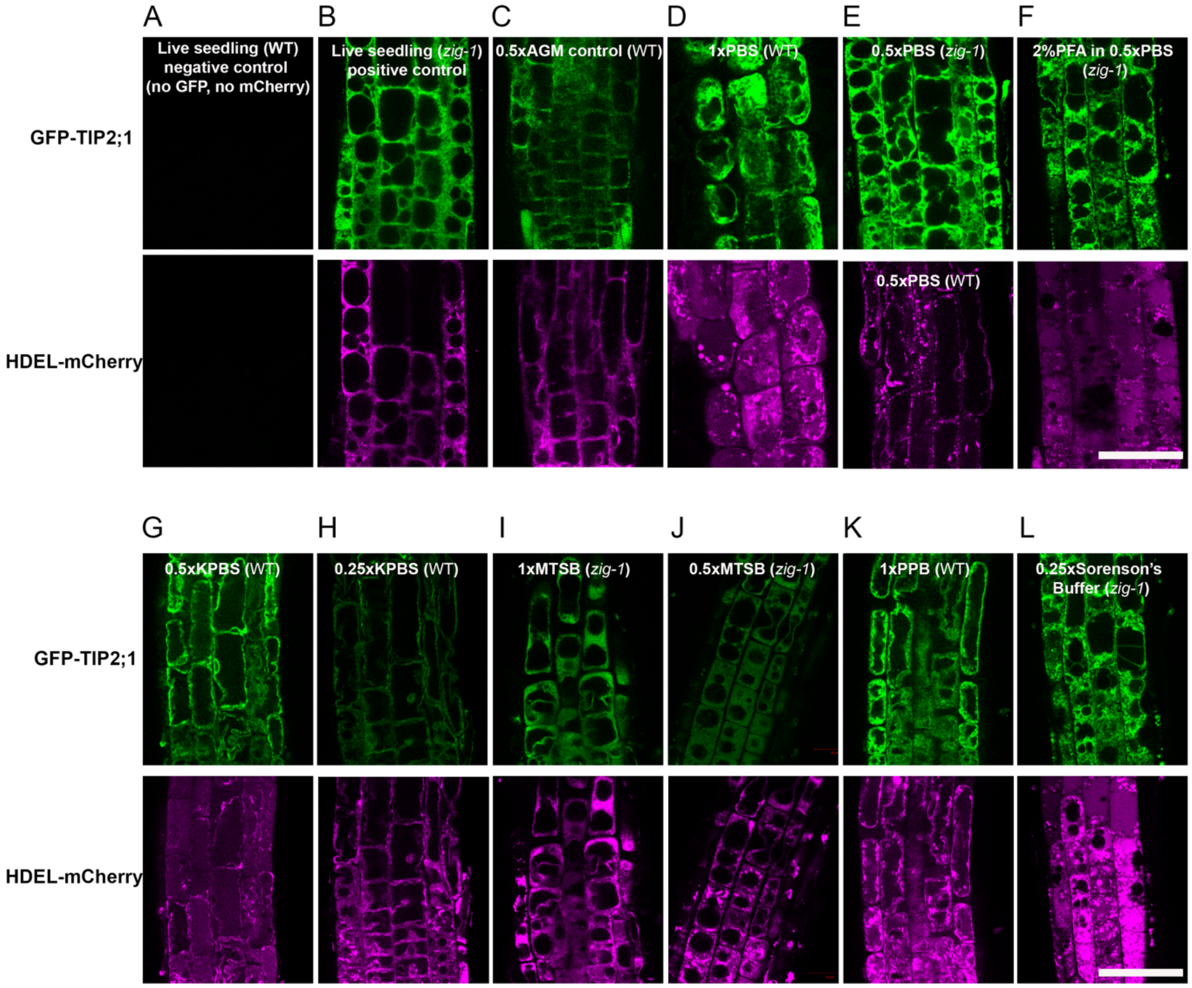

Supplementary figure 3. Comparison of different buffers for plant specimen fixation. Three-day-young seedlings from WT (**A, C, D, G, H, K**) or *zig-1* (**B, E, F, I, J, L**) grown in dark were first incubated in 1 volume of 0.5X AGM for 2 hours in dark and later with one volume of each fixative solution. The final concentration of buffers used are indicated and the final fixative was 4% PFA (except for F). Confocal images were taken after overnight fixation at 4°C. Using 4 % PFA in 0.5X PBS buffer was the best solution to preserve GFP intensity and tonoplast morphology for root tip cells. ER morphology was difficult to preserve in most buffers.
Scale bar: 40 μm

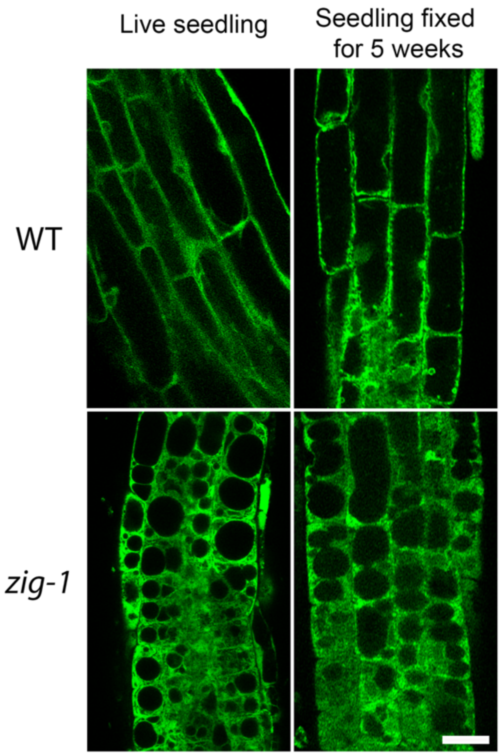

Supplementary figure 4. Samples can be stored at 4°C environment for 5 weeks after fixation with 4%PFA in 0.5X PBS.

Cell morphology and GFP signal of WT and *zig-1* expressing GFP-TIP2;1 can be well preserved as live seedlings after 5 weeks storage in fixative solution.

Scale bar: 20 μm

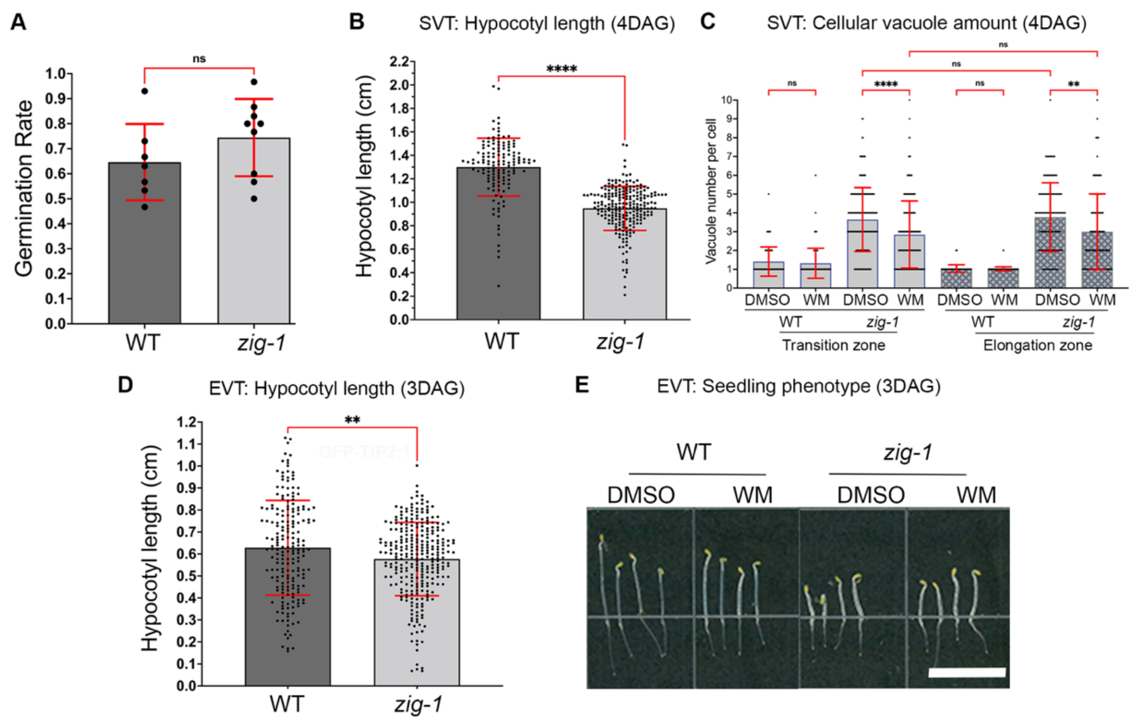

Supplementary figure 5. *Arabidopsis* WT and *zig-1* germinated and grew well in BRIC-PDFU from SVT and EVT.

(A) Germination rate of WT and *zig-1* grown in the BRIC-PDFU from SVT. No significant differences in germination rate were detected between genotypes (Unpaired t test, ns: p>0.05, n represents plate number, n>=7).

(B) Hypocotyl length of WT and *zig-1* grown in the BRIC-PDFU from SVT. WT showed longer hypocotyl than *zig-1* (unpaired t test, **** p<0.0001, n represents seedling number per genotype, n>135). Seedlings were grown at 22°C for 4 days (DAG: days after growth).

(C) Cellular vacuole number of WT and *zig-1* from SVT treated with DMSO buffer or WM solution. Cells from both the transition zone and the elongation zone of root tip were analyzed. (3-way ANOVA, Tukey's multiple comparison test, **** p<0.0001; **p<0.0021; n represents cell number under analysis per genotype per treatment, n>68)

(D) Hypocotyl length of WT and *zig-1* in the BRIC-PDFU from EVT. Seedlings were grown at 22°C for 3 days. (DAG: days after growth; Unpaired t test, ** p<0.0021, n represents seedling number per genotype, n>196). Raw datapoints are represented with filled black circles. Error bars represent standard deviation.

(E) Representative fixed seedlings of WT and *zig-1* in the BRIC-PDFU from EVT treated with DMSO buffer or WM solution before fixation.

Scale bar: 1 cm

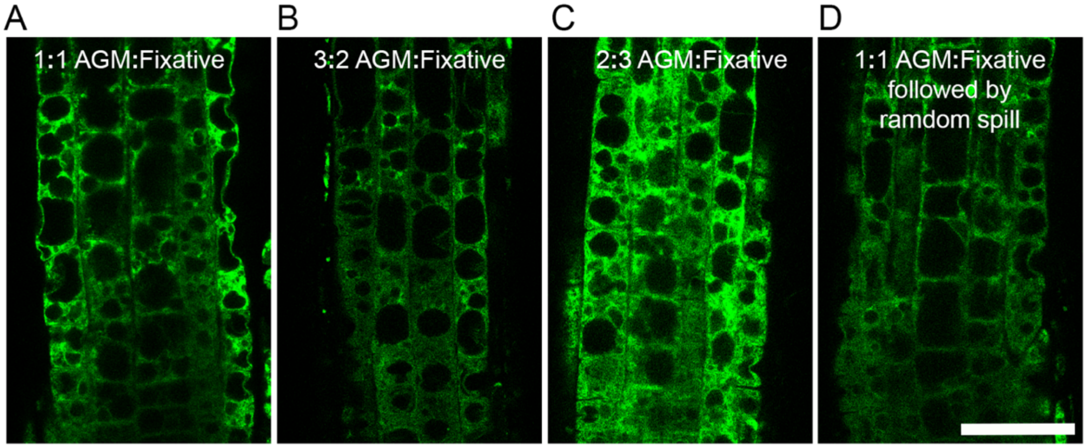

Supplementary figure 6. Root tip cells and GFP intensity were well preserved when the AGM and fixative solution were combined with 1:1 ratio.
Root tip confocal imaging for *zig-1* (GFP-TIP2;1) fixed in the AGM and fixative solution (8%PFA in 1X PBS) with a ratio of 1:1 (**A**); 3:2 (**B**); 2:3 (**C**) or 1:1 followed by solution spilling out from plate to mimic the similar liquid loss in the PDFU (**D**).
Arabidopsis seedlings were grown in dark for 3 days on petri dishes. Then 0.5X AGM was added to the petri dish for 2 hours in dark. Fixative solution was then added to the petri dishes to fix seedlings. Confocal images were taken after 4°C of overnight fixation.
Scale bar: 40 μm

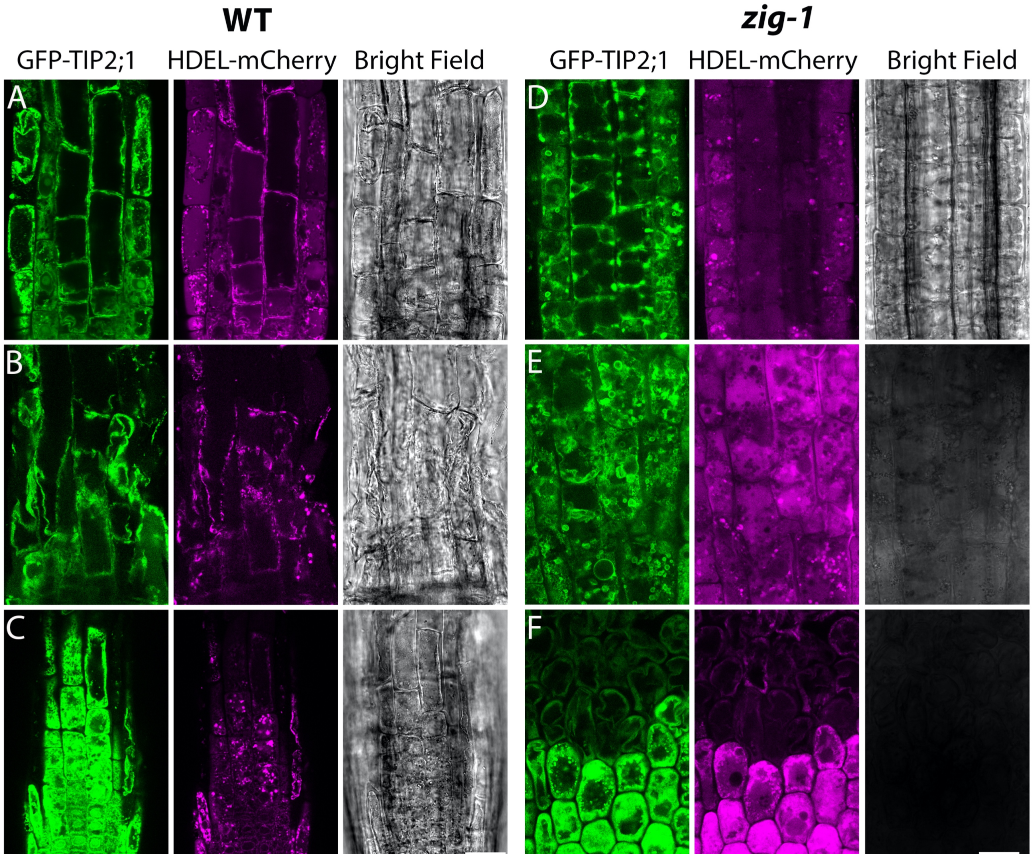

Supplementary figure 7. Examples of low-quality fixation specimens.
Fixation in the BRIC-PDFU did not always yield well-preserved seedlings. Examples of optimal specimens in WT **(A)** and *zig-1* **(D)** are shown. Examples of poorly fixed samples for WT **(B, C)** and *zig-1* **(E, F)** are included.
Scale bar: 20 μm

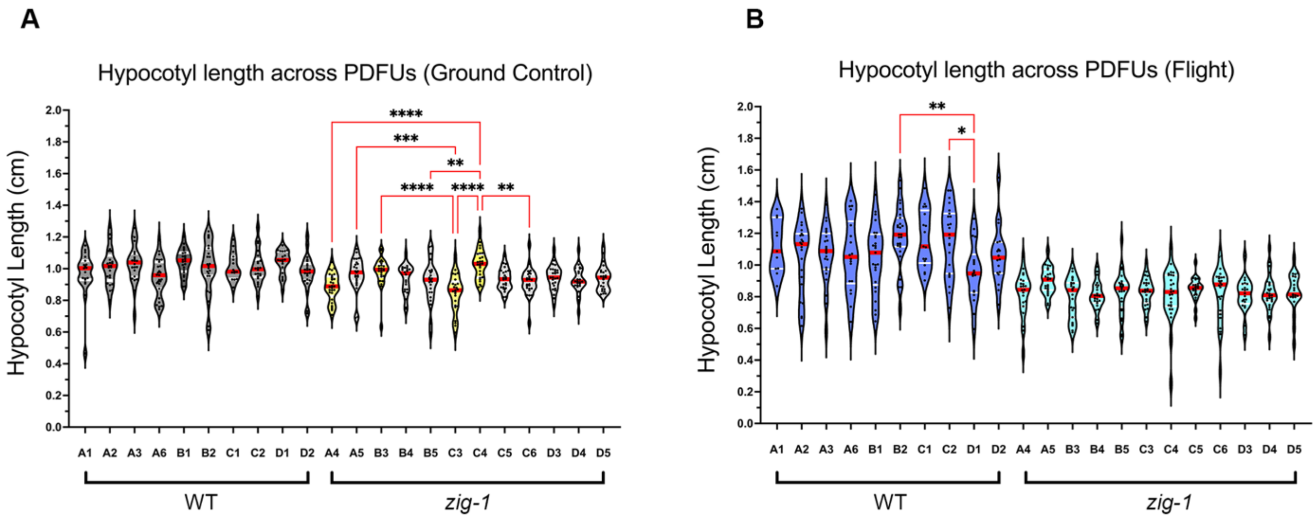

Supplementary figure 8. Data of hypocotyl length for WT and *zig-1* from ground control (**A**) and flight assay (**B**) were consistent across different PDFUs. Violin plots depicting raw data points (filled black circles), median (red line), quartiles (white lines) were shown for individual PDFUs. The bottom and top data points represent the lower and upper adjacent values respectively. N: 19-29 hypocotyls per PDFU.

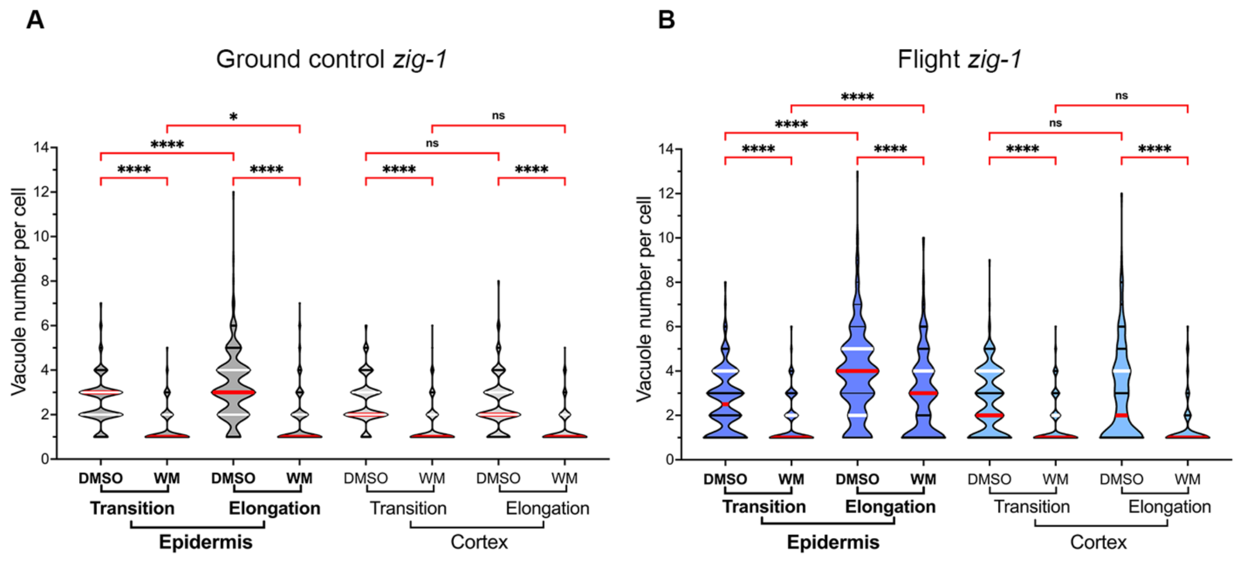

Supplementary figure 9. Cells from the epidermis transition zone and the elongation zone had different vacuole numbers while cells from the cortex transition zone and the elongation zone had similar vacuole numbers when considering *zig-1* samples from ground control (**A**) and flight assay (**B**). Violin plots depicting raw data points (filled circles that appear as lines due to the number of data points), median (red line), quartiles (white lines) were shown. The bottom and top data points represent the lower and upper adjacent values respectively. N: 150-270 cells per treatment.

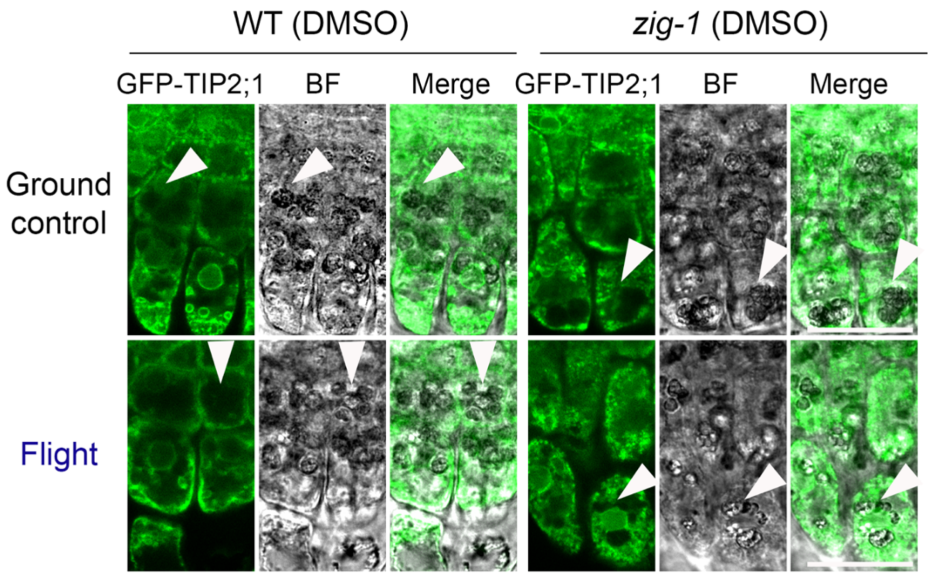

Supplementary figure 10. Visualization of amyloplast in columella cells.
Amyloplasts (Arrowhead) can be observed in the root tip columella cells for WT and *zig-1* that were grown in the BRIC-PDFU of ground control and flight assay.
Scale bar: 20μm
